# Validation of a novel stand-alone software tool for image guided cardiac catheter therapy

**DOI:** 10.1007/s10554-019-01541-9

**Published:** 2019-01-28

**Authors:** René van Es, Hans T. van den Broek, Mira van der Naald, Leon de Jong, Eliane R. Nieuwenhuis, Adriaan O. Kraaijeveld, Pieter A. Doevendans, Steven A. J. Chamuleau, Frebus J. van Slochteren

**Affiliations:** 1 Department of Cardiology, Division Heart and Lungs, University Medical Center Utrecht, PO Box 85500, 3508GA Utrecht, The Netherlands; 2 MIRA Institute for Biomedical Engineering and Technical Medicine, University of Twente, Enschede, The Netherlands; 3 Netherlands Heart Institute, Utrecht, The Netherlands; 4 RMU, Regenerative Medicine Center Utrecht, Utrecht, The Netherlands

**Keywords:** Hybrid imaging, Innovation, MRI, Myocardial viability imaging, Pre-clinical research, Image guided therapy

## Abstract

Comparison of the targeting accuracy of a new software method for MRI-fluoroscopy guided endomyocardial interventions with a clinically available 3D endocardial electromechanical mapping system. The new CARTBox2 software enables therapy target selection based on infarction transmurality and local myocardial wall thickness deduced from preoperative MRI scans. The selected targets are stored in standard DICOM datasets. Fusion of these datasets with live fluoroscopy enables real-time visualization of MRI defined targets during fluoroscopy guided interventions without the need for external hardware. In ten pigs (60–75 kg), late gadolinium enhanced (LGE) MRI scans were performed 4 weeks after a 90-min LAD occlusion. Subsequently, 10–16 targeted fluorescent biomaterial injections were delivered in the infarct border zone (IBZ) using either the NOGA 3D-mapping system or CARTBox2. The primary endpoint was the distance of the injections to the IBZ on histology. Secondary endpoints were total procedure time, fluoroscopy time and dose, and the number of ventricular arrhythmias. The average distance of the injections to the IBZ was similar for CARTBox2 (0.5 ± 3.2 mm) and NOGA (− 0.7 ± 2.2 mm; p = 0.52). Injection procedures with CARTBox2 and NOGA required 69 ± 12 and 60 ± 17 min, respectively (p = 0.36). The required endocardial mapping procedure with NOGA prior to injections, leads to a significantly longer total procedure time (p < 0.001) with NOGA. Fluoroscopy time with NOGA (18.7 ± 11.0 min) was significantly lower than with CARTBox2 (43.4 ± 6.5 min; p = 0.0003). Procedures with CARTBox2 show a trend towards less ventricular arrhythmias compared to NOGA. CARTBox2 is an accurate and fast software-only system to facilitate cardiac catheter therapy based on gold standard MRI imaging and live fluoroscopy.

**Electronic supplementary material:**

The online version of this article (10.1007/s10554-019-01541-9) contains supplementary material, which is available to authorized users.

## Introduction

Multimodality image integration during fluoroscopy guided cardiac interventions enables conjunct visualization of soft tissue characteristics during the intervention. X-ray fluoroscopy (XRF) is traditionally used to guide the catheter during minimally invasive procedures. Since XRF is unable to accurately display soft tissues, fusing XRF with MRI images enables superimposition of the cardiac anatomy on fluoroscopic images [1, 2]. Since the contrast of native clinical MRI scans can be low, the benefit of solely anatomy visualization is limited and does not allow the user to determine exact targets. A real benefit arises when the MRI dataset is pre-processed before the fusion to emphasize and visualize only its relevant anatomical of functional aspects during the procedure.

To optimize procedural success accurate identification of the target location for e.g. ablation of an arrhythmic focus, the positioning of a pacing lead or performing an endomyocardial biopsy or injection, it is important that identification is done based on the associated gold standard imaging modality. In close collaboration with CART-Tech B.V. (Utrecht, the Netherlands), we have therefore developed CARTBox2 software, which facilitates offline MRI based treatment planning for cardiac catheter interventions. CARTBox2 is MRI-vendor, XRF-vendor and catheter-vendor independent and enables the user to process a MRI dataset to annotate and visualize parameters of interest such as myocardial infarction transmurality or myocardial wall thickness. The software subsequently stores all information in a DICOM treatment dataset which contains the annotated information as well as anatomical data to facilitate its registration with XRF. The annotated regions can then be visualized in conjunction with the interventional fluoroscopy imaging system in real-time during the intervention.

The aim of the present study was to assess the accuracy of CARTBox2 for the targeted delivery of cardiac catheter therapy to the border zone of a myocardial infarction based on the gold standard for myocardial infarction assessment: Late Gadolinium Enhancement (LGE)-MRI. For comparison the clinical standard NOGA® XP platform (Biosense Webster Cordis, Johnson & Johnson, USA) for intramyocardial injections in the infarct border zone (IBZ) based on electro-mechanical maps was used [3]. In a porcine model of myocardial infarction, we compared the IBZ targeting accuracy of the CARTBox2 and the NOGA system. Secondary endpoints were the total XRF time and dose, procedure time and arrhythmogenicity of the procedure.

## Methods

### Animals

All experiments were performed in accordance with the “Guide for the Care and Use of Laboratory Animals”, prepared by the Institute of Laboratory Animal Resources, and with prior approval by the Animal Experimentation Committee of the Faculty of Medicine, Utrecht University, the Netherlands. For this study, 14 6-months old 60–75 kg female Dutch Topigs pigs (Van Beek SPF varkensfokkerij B.V., Lelystad, The Netherlands) were subjected to an antero-septal myocardial infarction by a 90-min occlusion of the left anterior descending artery distal to the second diagonal branch, according to a previously described protocol [4]. Pre-treatment and anesthesia during the ischemia/reperfusion (I/R) procedure were performed as described before [5]. Before starting all procedures, 300 mg amiodarone and heparin (100 IE/kg after positioning the sheaths and 50 IE/kg every 2 h) were administered. Pigs were mechanically ventilated with a positive pressure ventilator with FiO_2_ = 0.5, 10 ml/kg tidal volume and a frequency of 12/min under continuous capnography. An additional arterial line was inserted for continuous arterial blood pressure monitoring. After I/R, the animals were housed in stables for 4 weeks.

**Fig. 1:**
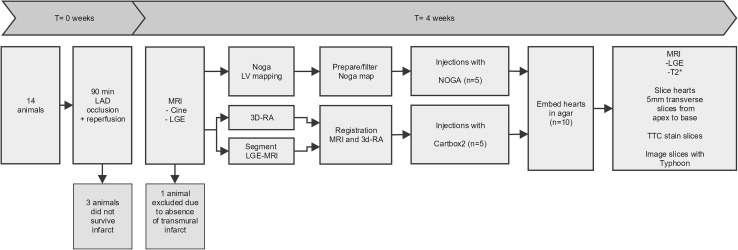
Experimental study design. A total of 14 animals were subjected to a myocardial infarction, ten animals underwent the injection procedure. Five animals were randomized into the NOGA-arm, whilst 5 animals were randomized to the CARTBox2 group. All ten hearts were embedded and analyzed

Before the injection procedure, 4 weeks after I/R, all animals underwent an LGE-MRI scan to visualize the myocardium, the myocardial wall thickness, the myocardial infarction and IBZ (MRI parameters are listed in the Supplementary Data). For the injections, a mixture of 10% ureido-pyrimidinone gel, super paramagnetic iron oxide (SPIO) particles (15 µg/ml; Sinerem, Guerbet, France) and fluorescent beads (10.000 beads/ml, 580/em605, Molecular Probes Invitrogen) was used [6–8]. The animals were randomized to undergo intramyocardial injections using either the NOGA system or CARTBox2 (Fig. 1). In each animal at least 10 injections (0.2 ml) were performed using the Myostar® injection catheter (Biosense Webster), needle extension was set to half of the myocardial wall thickness in the IBZ measured on MRI. After the injection procedures, gadolinium was administered intravenously (GadoVist 0.2 ml/kg) and 15 min later the animals were euthanized using an intravenous bolus of 20 ml 7.5% potassium chloride.

### Injection procedure

#### The NOGA group

The NOGA system was used to create an electromechanical map of the left ventricle (LV) using the NOGAStar® mapping catheter that provides 3D magnetic tracking [9]. To create the 3D Map the catheter is steered over the LV endocardial wall to measure local unipolar and bipolar depolarization voltages and wall deformation (linear local shortening). By acquiring the end diastolic positions of the catheter during the measurements (mapping points) on the LV endocardium a 3D point cloud is created [10]. Subsequently, the measured parameters are interpolated between the points to reconstruct a 3D endocardial surface on which the location of the infarct, the IBZ, and consequently the target area for the injections can be identified based on the measured parameters. After the mapping procedure a trackable injection catheter is inserted into the LV to perform injections in to the IBZ based on the created 3D map.

#### Preparation

An 8F sheath was placed in the right femoral artery. Under XRF guidance, the NOGAStar^®^ mapping catheter (Biosense Webster) was introduced into the LV via retrograde passage through the aortic valve. Using the NOGA system (SW version 1.1.43), measurements were performed homogeneously over the endocardial wall to ensure complete al coverage of the LV endocardium. After the mapping procedure, the NOGA maps were filtered using the moderate filter option of the NOGA system. If necessary more points were acquired to maintain the homogeneous distribution of the points over the endocardium. The map was finalized when at least 100 points met the aforementioned criteria. Subsequently the infarct was identified by setting bipolar voltages maps to a range of 0.5–1.5 mV, and a target line (preferred injection location) was manually drawn on the 1 mV area around the infarct using the NOGA system software.

#### Injection procedure

The Myostar® injection catheter was introduced into the LV and injections were performed at the target line. In both study groups, a provoked extra-systole on the ECG signal upon needle exertion was defined as successful needle insertion in the myocardium.

### CARTBox2 group

#### Preparation

**Fig. 2:**
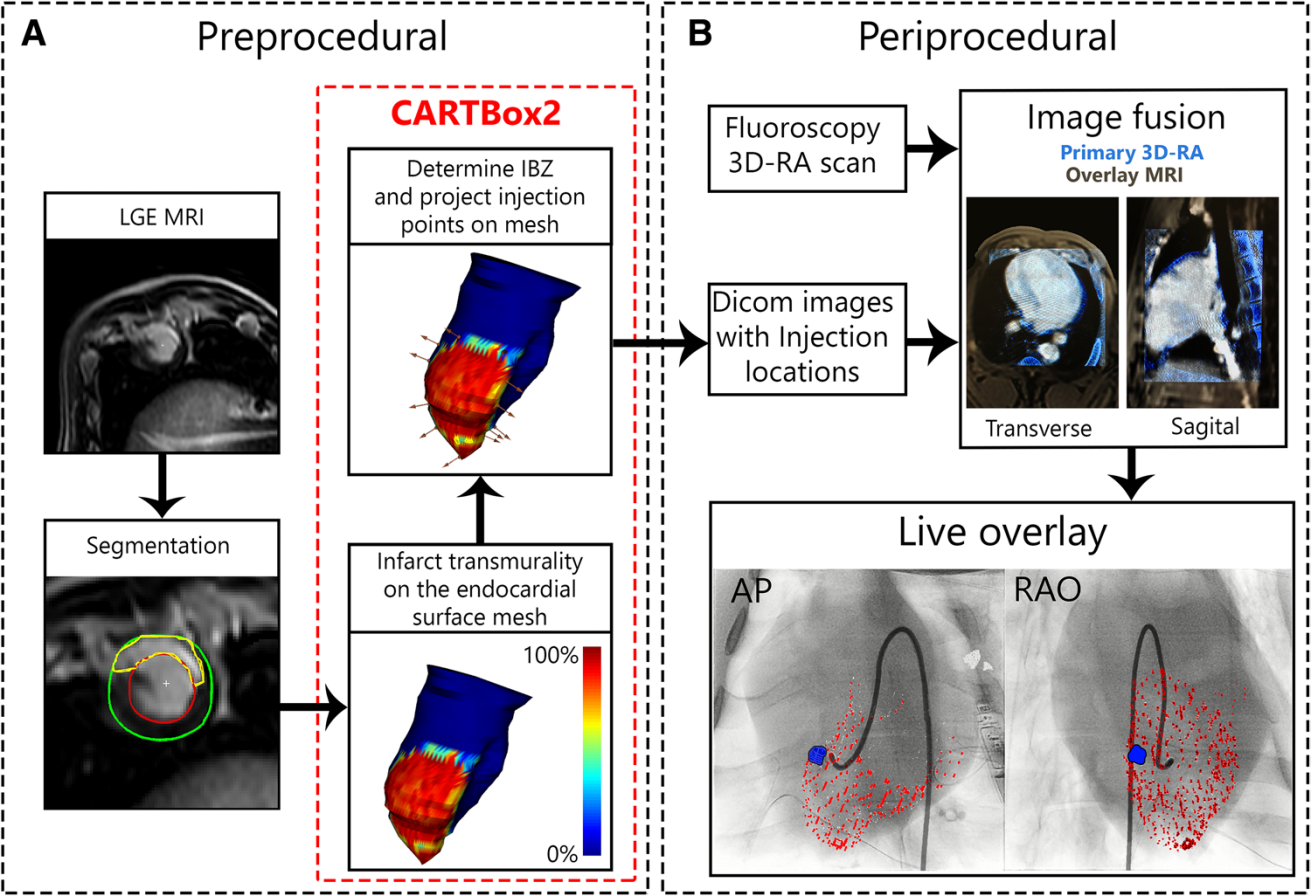
CARTBox2 workflow. **a** The preprocedural steps include the acquisition of an LGE-MRI scan of the left ventricle. The endocardium, epicardium and infarct are then segmented. Using CARTBox2, from these segmentations, the scar transmurality is calculated and projected onto the endocardial surface mesh. Subsequently, the IBZ is calculated and the injection locations are defined and projected onto the mesh (brown arrows), these locations are embedded into the MRI DICOM images. **b** After acquiring a 3D-RA scan, the MRI DICOM image is fused with the 3D-RA image based on skeletal anatomy. During the injection procedure, the target locations are visualized on the live fluoroscopic images. The AP and RAO images showing the endocardium (red) and target (blue) are visually enhanced for printing purposes. *LGE-MRI* late gadolinium enhanced magnetic resonance image, *IBZ* infarct border zone, *3D-RA* 3-dimensional rotational angiography, *AP* anterior-posterior, *RAO* right anterior oblique

First, endo- and epicardial contours were manually segmented in the end-diastolic LGE-MRI images using the freely available software Segment version 2.0 R4265 (http://segment.heiberg.se) [11]. Subsequently, the infarct was segmented with Segment based on the full width at half maximum algorithm (Fig. 2) [12, 13]. Next the segmentations were loaded into the CARTBox2 software to assign 16 injection targets into the IBZ region with 1–20% infarct transmurality and wall thickness (WT) of > 5 mm, equally distributed over the septal and anterior side of the infarct. Areas with transmurality higher than 20% or WT < 5 mm were marked as danger zones. Thereafter, the targets were stored in a separate DICOM treatment dataset for each target. The infarct transmurality (e.g. with 20% infarct transmurality, 20% of the myocardial in the radial direction consists of infarcted myocardium) was determined using the full width at half maximum segmented LGE-MRI images [12, 13].

#### Injection workup

A 3D rotational angiography (3D-RA) roll scan of the thorax was performed using a single plane C-arm (Allura FD 20, Philips Healthcare, the Netherlands; parameters in Supplementary Data). A 3D reconstruction is made from a 180 degrees rotation of the C-arm during 10.2 s. With an average heartrate of 60 bmp during the rotational scan on average 10 heartbeats are contained in the 3D reconstruction leading to a 3D representation of the end diastolic outer contour of the heart. The DICOM treatment datasets containing the injection targets are also acquired at end diastole and were semi-automatically fused with the resulting 3D-RA dataset, first based on thoracic anatomy to alight the two volumes and secondly based on the epicardial contour using Interventional Tools (R8.8.1) on the Philips workstation. Meanwhile, an 8F sheath was placed in the right femoral artery.

#### Injection procedure

The Myostar® injection catheter was introduced into the LV via femoral access and retrograde passage through the aortic valve under XRF guidance. The injection targets were displayed in combination with XRF. After registration, the targets are locked to the XRF hardware and move in conjunction with the XRF detector/C-arm movement. Injections were aimed at the targets displayed on XRF. Targeting of a displayed point was verified by inspecting the catheter location from at least 2 fluoroscopic angles that were at least 30 degrees apart.

### Histological processing

After euthanasia, the heart was excised and prepared in an agarose solution for coarse histological analysis as previously described [14].

After complete gelation of the agarose, ex vivo LGE and T2* MRI scans were performed to image the SPIOs (parameters in Supplementary Data). Thereafter, the embedded heart was cut into 5 mm thick transversal slices starting at the apex with a Berkel® meat slicer. The cuts were angulated identical to the angulation of short-axis ex-vivo MRI acquisitions. Tissue slices were stained in a 1 m% dissolved 2,3,5-triphenyltetrazoliumchloride (TTC) solution. Both sides of every slice were photographed and scanned using a Typhoon 9410 (GE Healthcare) variable mode scanner set at 2 channels (ch.1 532 nm, filter: 580BP30; ch.2 633 nm, filter: 610BP30) with an image resolution of 0.1 × 0.1 mm to visualize the fluorescent beads.

## Data analysis

### Per-procedural ventricular arrhythmia monitoring

In 8 animals ECG leads of a Holter device (Fysiologic ECG Services, Amsterdam, The Netherlands) were attached to the chest of the animals to enable continuous recording during the mapping and injection procedures. The total number of premature ventricular contractions (PVCs), non-sustained ventricular tachycardias (VT) and sustained VTs were counted during the mapping procedure and each separate injection by two investigators blinded for randomization. A PVC was defined as a single ectopic complex, a doublet was counted as 2 PVCs. Non-sustained VTs were defined as 3 or more consecutive ventricular complexes with a frequency more than 100/min with a total duration shorter than 30 s.

### Injection accuracy assessment

In the fluorescent images, the myocardial scar tissue was segmented and the TTC and ex vivo LGE-MRI images were used for verification of the infarct location and morphology [14]. The positions of the injections were annotated in the fluorescence images. The primary endpoint of this study, the distance of each injection to the 1–20% infarct transmurality region was measured along the endocardial contour in the segmented fluorescence images by two observers blinded for study randomization (Fig. 4). The depth of each injection and the wall thickness were measured perpendicular to the endocardial contour.

### Statistical analysis

All normally distributed data are presented as mean ± standard deviation. A Shapiro–Wilk test was used to test the data for normality. With all repeated measurements, a mean per animal was used for comparison. Continuous variables with a normal distribution were compared with a two tailed unpaired *t* test, categorical data was compared using a Chi square test. No-outlier analysis was performed. A p-value of < 0.05 was considered to be statistically significant. All analyses were performed using SPSS (IBM® SPSS® Statistics, version 23).

## Results

An antero-septal myocardial infarction was induced successfully in 14 animals. One animal died during the infarct induction, one animal died in the night following infarct induction, most likely due to a cardiac arrhythmia. One animal was euthanized 1 week after infarct induction after reaching the ‘humane’ end-point due to congestive heart failure. Eleven animals survived to 4 weeks (Fig. 1). One animal was excluded from the study after the MRI scan due to absence of a clear infarct and IBZ. Consequently, in total 5 animals were treated and analyzed in both groups. Baseline characteristics were balanced between the two study groups (Table 1).

**Table 1:** Baseline characteristics

Parameter	NOGA (n = 5)	CARTBox2 (n = 5)	p-value
Body weight (kg)	72.7 ± 6.4	70.5 ± 5.1	0.559
LV mass (gr)	132 ± 14	120 ± 19	0.309
Infarct size (gr)	13.4 ± 5.0	19.0 ± 5.8	0.141
Ejection fraction (%)	52.6 ± 8.7	46.9 ± 4.4	0.236
End diastolic volume (ml)	127 ± 27	126 ± 27.5	0.950
End systolic volume (ml)	61 ± 20	67 ± 17	0.658

All parameters except body weight were measured using MRI

### Noga mapping procedure

In the 5 animals that underwent electromechanical mapping with the NOGA system, an average of 135 ± 57 measurements (points) were acquired per map. The average NOGA mapping procedure duration was 90.0 ± 9.2 min. The average XRF time and dose during mapping were 12.9 ± 9.9 min and 18.2 ± 19.0 Gy cm^2^, respectively. A total of 157 ± 62 PVCs and 78 ± 56 non-sustained VTs were observed during the mapping procedure.

### Intramyocardial injections

**Fig. 3:**
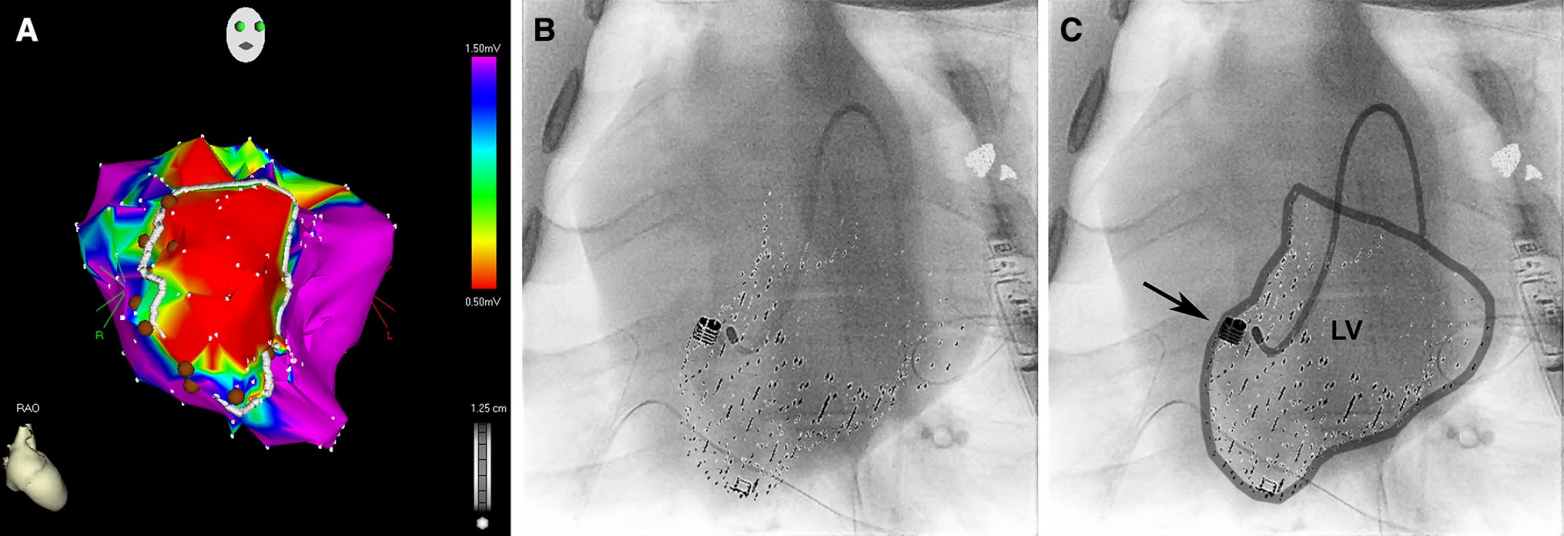
Examples of NOGA and CARTBox injection procedure. **a** Example of a NOGA bipolar voltage map (color scale: 0.5–1.5 mV) in right anterior oblique view, with design line (white) to indicate the infarct border zone. The injections are shown as brown circles. **b** Anterior-posterior X-ray fluoroscopy image fused with the CARTBox2 modified MRI scan. The left ventricular endocardium (small dots) and the injection target (large dot). **c** The same image as shown in **b**, the endocardial wall and catheter are visually enhanced. The arrow indicates the injection target defined on late gadolinium enhanced MRI

**Fig. 4:**
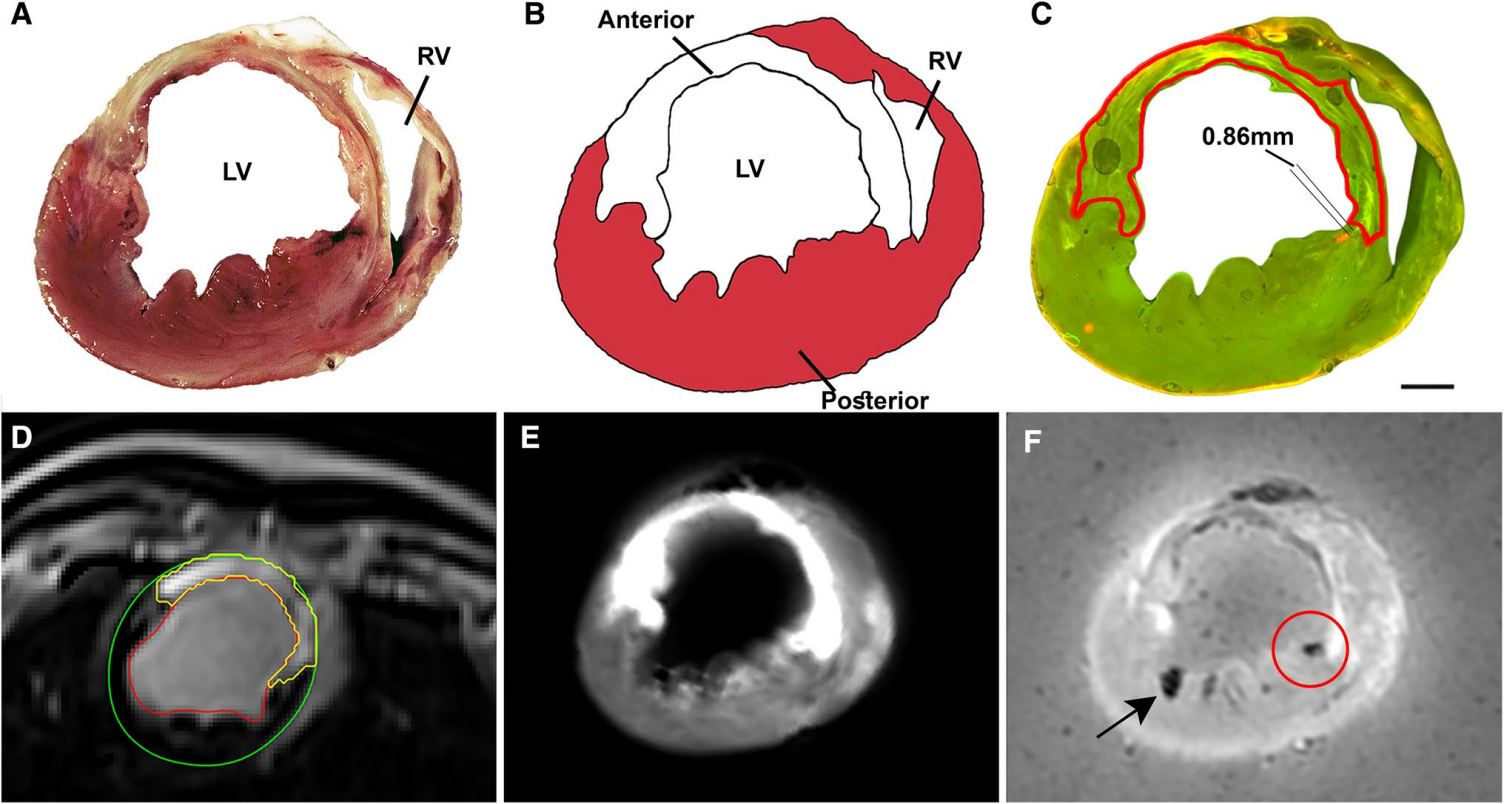
Example of histological analysis. **a** Photograph of a heart slice after TTC staining. **b** Schematic impression of the heart slice shown in **a**, with the myocardium shown in red and the infarct in white. **c** Fluorescent image of the same heart slice showing the injection deposition in orange. The infarct segmentation is shown in red. The parallel lines indicated the measured distance from injection to the infarct area. **d** In vivo LGE-MRI scan of the heart. The segmentation was used in CARTBox2, the red line = endocardial border, green line = epicardial border and yellow line = infarct segmentation. **e** Ex vivo LGE-MRI image of the heart slice shown in figures **a**–**c**, which was used to segment the infarct location. **f** Ex vivo T2* image of the heart slice that was used to confirm the location of the injections found with fluorescent imaging (indicated by red circle). The arrow points towards an air-bubble

**Fig. 5:**
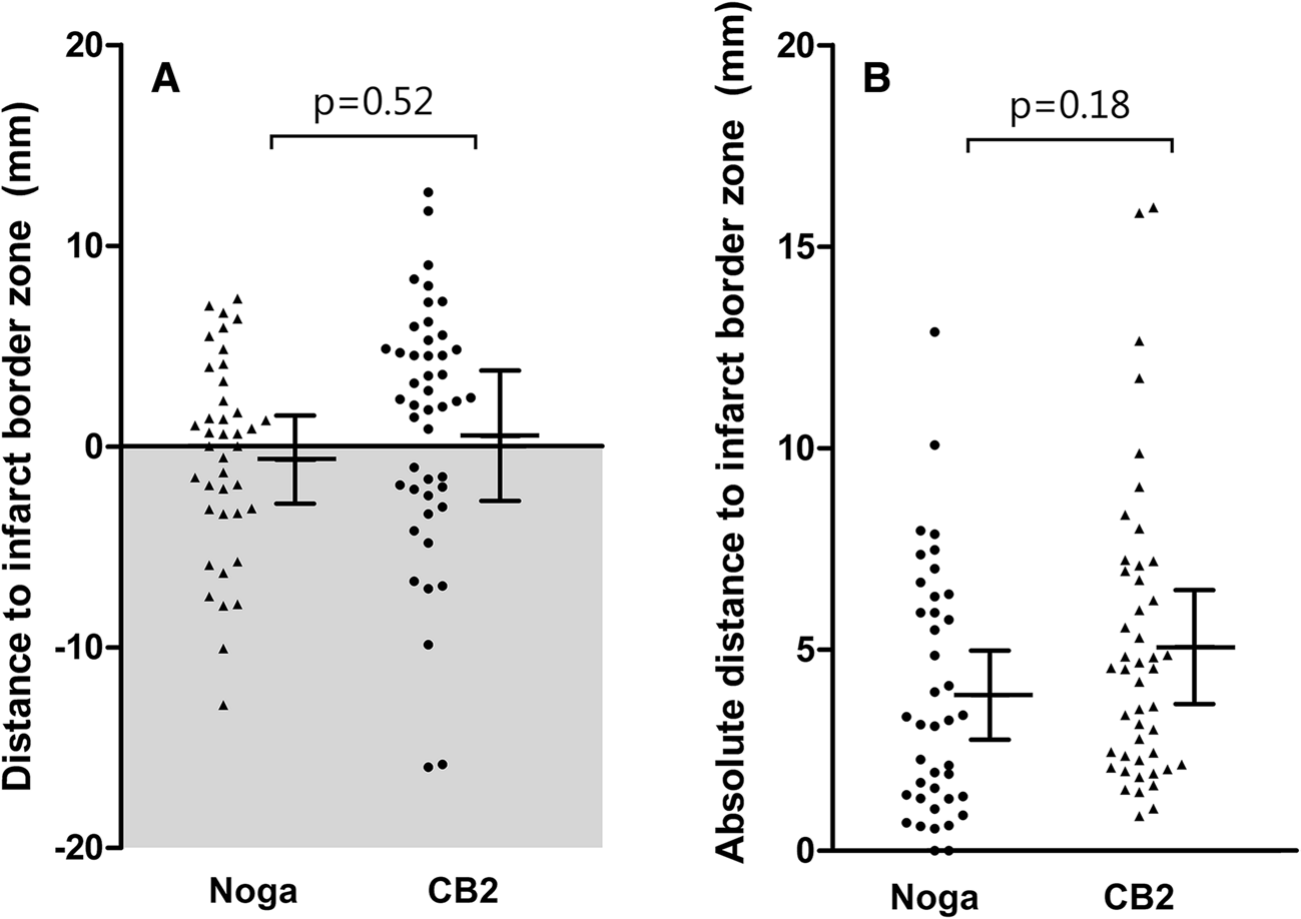
Details of injections. **a, b** The dots represent individual injections with NOGA (n = 40) and CARTBox2 (n = 46). The error bars show the mean and standard deviation of the injections in each animal. **a** The gray area indicates the infarct area

A total of 118 injections were performed using the NOGA system (5 animals, 58 injections; e.g. Fig. 3a) or the CARTBox2 system (5 animals, 60 injections; e.g. Fig. 3b, c). During histological analysis, 8.0 ± 1.2 (71 ± 16%) and 9.2 ± 3.1 (76 ± 16%; p = 0.45) injections were retrieved in the NOGA and CARTBox2 groups, respectively (e.g. Fig. 4). The average distance of the injections to the IBZ was not significantly different for NOGA (− 0.7 ± 2.2 mm) and CARTBox2 (0.5 ± 3.2 mm; p = 0.52) (Fig. 5). The average injection depth was 2.9 ± 1.5 mm and 3.4 ± 1.0 mm (p = 0.59) for NOGA and CARTBox2, respectively.

### Injection procedure duration

The total procedure duration of CARTBox2 (69.2 ± 11.9) was significantly shorter than the total procedure duration of NOGA (150.2 ± 12.4) (p < 0.001) (Table 2). The CARTBox procedure contains the 3D-RA and injection procedure whereas the NOGA procedure contains both a mapping and an injection procedure (Table 2). No significant difference was found in the duration of the injection parts of the two methods: 69.2 ± 11.9 vs. 60.2 ± 16.7 for CARTBox and NOGA, respectively (Table 2).

**Table 2:** Injection procedure parameters

	NOGA (n = 5)	CARTBox2 (n = 5)	p
Injection procedure			
Time (min)	60.2 ± 16.7	69.2 ± 11.9	0.355
Total XRF time (min)	6.1 ± 6.2	43.4 ± 6.5	< 0.001
Total DAP (Gy·cm^2^)	11.2 ± 18.0	64.2 ± 49.0	0.071
PVCs (#)^a^	239 ± 100	283 ± 10	0.42
Non-sustained VTs (#)^a^	41.5 ± 11.7	74.3 ± 35.0	0.13
Per injection			
Time (min/injection)	5.4 ± 1.8	6.0 ± 1.4	0.55
Total XRF time (min/injection)	0.5 ± 0.5	3.7 ± 0.8	< 0.001
Total DAP (Gy·cm^2^/injection)	0.9 ± 1.4	5.4 ± 3.6	0.031
PVCs (#)^a^	21.0 ± 7.3	20.3 ± 1.1	0.86
Non-sustained VTs (#)^a^	3.7 ± 0.5	5.2 ± 2.1	0.21
Total procedure (Mapping/3D-RA + injections)			
Total time (min)	150.2 ± 12.4	69.2 ± 11.9	< 0.001
Total XRF time (min)	18.7 ± 11.0	43.4 ± 6.5	0.003
Total DAP (Gy·cm^2^)	29.4 ± 35.7	69.8 ± 49.0	0.174
PVCs (#)^a^	396 ± 138	283 ± 10	0.20
Non-sustained VTs (#)^a^	120 ± 68	74.3 ± 35.0	0.28

*DAP* dose area product, *XRF* X-ray fluoroscopy, *PVC* premature ventricular contraction, *VT* ventricular tachycardia

^a^Holter recording and analysis were performed only in the last 8 animals (4 in each group)

### XRF time and dose during injections

In the CARTBox2 group, the average 3D-RA roll scan radiation dose was 5.6 ± 1.6 Gy·cm^2^. Significantly more XRF time and dose was used with the CARTBox2 procedures (Table 2).

### Ventricular arrhythmia monitoring

In both groups Holter recordings were successfully analyzed in four animals. In the NOGA and CARTBox2 groups, respectively 2 and 1 animal(s), suffered a sustained VT during the injection procedure that required cardioversion. Procedures with CARTBox2 showed a trend towards fewer PVCs and non-sustained VTs than NOGA procedures (Table 2).

## Discussion

In this study we have assessed the accuracy of CARTBox2 to target injections to the IBZ in a large animal model of myocardial infarction. For comparison we have used a clinically available electromechanical mapping and catheter tracking system dedicated to perform intramyocardial injections into the IBZ. To the best of our knowledge, this is the first study in which an MRI-vendor, XRF-vendor and catheter-vendor independent software-only image fusion technique was used for the real-time visualization of injection targets during intramyocardial injections performed with XRF. We showed that CARTBox2 is able to accurately guide intramyocardial injections to the targets located on the IBZ. Since CARTBox2 does not require an endocardial mapping procedure, CARTBox2 procedures were significantly shorter than procedures performed with NOGA. Because navigation with CARTBox2 is done solely based on XRF, the total XRF time and dose were significantly higher with CARTBox2 procedures than with NOGA, but are comparable with a typical percutaneous coronary intervention (PCI). The number of induced cardiac arrhythmias showed a trend towards being lower in the CARTBox2 group.

### Pig model

The myocardial infarction induced by 90 min LAD occlusion lead to severe myocardial infarctions with a clear IBZ, which facilitates clear measurement of the primary endpoint (Fig. 4). All unexpected deaths occurred before interventions were performed and can therefore only be attributed to the severity of the myocardial infarctions. The exclusion of one pig due to small non transmural infarction can probably be attributed to non-total occlusion of the LAD after a resuscitation session during the infarct induction procedure.

### Accuracy

With CARTBox2 we were able to define the injection target zone based on the gold standard imaging modality for myocardial infarction transmurality and wall thickness. In this study the target region in the CARTBox2 group was set at 1–20% infarct transmurality and > 5 mm wall thickness to assure histological identification of the IBZ and to avoid the transmurally infarcted and thin myocardium. In both study groups, the injections were performed using the 8F MyoStar® injection catheter. In both groups, the histological retrieval rate of the injections in both groups was not 100%, (range 54–93%). This can be explained by (1) non-successful injections, e.g. an extra-systole was provoked upon needle excursion, but the biomaterial was not delivered into the tissue, and (2) the penetration depth of the fluorescent light was limited to 1.5 mm while the histological slices were cut at 5 mm to allow tissue handling. On average, injections performed with NOGA were located on the inside of the IBZ (-0.7 ± 2.2 mm), while injections with CARTBox2 were located on the outside of the IBZ (0.5 ± 3.2 mm; p = 0.52). This difference is most likely caused by the slightly different region that is targeted by the set 1–20% infarct transmurality zone with CARTBox2 compared to the 0.5–1.5 mV setting with NOGA. Despite some studies showed considerable variability in bipolar voltage (BV) values for regions of specific infarct transmurality it was also shown that BV was specifically sensitive for transmural infarcts [10, 15]. BV is therefore considered the best parameter to assess infarct tissue since it is less sensitive for far-field signals [15, 16]. The thresholds used in this study were selected based on extensive literature study and historical data. Since the injections in both study groups are placed close to the real IBZ we believe that the error induced hereby was minimal. To further limit this intrinsic error of the NOGA procedure we performed extensive mapping and applied filtering so that at least 100 points homogeneously distributed over the LV endocardium remained in each dataset to minimize the error introduced by interpolation of endocardial potentials and point locations.

With CARTBox2 procedures a registration error between the LGE-MRI and 3D-RA scan can lead to a targeting error for all injections in a procedure. Theoretically, the total registration error consists of a combination of errors introduced in the multiple steps involved in the protocol, LGE-MRI segmentation, 3D-RA reconstruction and in the registration of the LGE-MRI scan with the 3D-RA. The accuracy of the segmentation of the MRI and reconstruction of the 3D-RA is limited by the resolution of the acquired data. The accuracy of the registration of both image modalities was not evaluated in this study, however by using a step-wise registration method based on skeletal and cardiac anatomy the chance of registration errors is minimized. As a proof thereof we did not observe structural differences of the location between the anterior and septal injections in the CARTBox2 groups, suggesting that a structural shift during registration was not present.

In this study, XRF during the CARTBox2 injection procedure was displayed at 3.75 frames/s, while the overlay consisted of a static end-diastolic endocardial posture of the heart. While navigating to the targets, the operator had to interpret the end-diastolic endocardialcatheter position in each cardiac cycle. Using the two XRF-angle approach, the operator was however able to accurately assess whether the catheter reached a target position. A large difference in the location of the endocardium between the LGE-MRI and 3D-RA, for example due to a difference in the hemodynamic filling state of the subject should be avoided to ensure accurate navigation.

With CARTBox2, fusion of LGE-MRI images with XRF is performed on the XRF workstation provided by the XRF vendor and therefore is state of the art, and does not require external hardware during the injection procedure. Other studies have used online image fusion systems to guide intramyocardial injections that run on an external computer connected to the XRF system to fuse it with LGE-MRI,[2] CT or 18F-FDG-PET/CT images [1]. Comparing the reported accuracy for LGE-MRI based XRF interventions of these respective studies 0.9 ± 5.0 mm (mean ± SD) and 4.8 ± 0.5 mm (mean ± SEM) and with our data 0.5 ± 3.2 mm (mean ± SD), suggests that the IBZ injection accuracy of CARTBox2 is in the same order of magnitude.

### Procedure duration

Intramyocardial injections performed with the NOGA system require the creation of a detailed endocardial surface map. Average mapping time in this study was 90 min whereas a typical clinical NOGA mapping procedure takes about 45 min [10]. The double time taken for the mapping procedure in this study can be attributed to the chosen primary study endpoint, which required detailed mapping and filtering of the electro anatomical map to optimally assess the IBZ. The total procedure time, including the mapping and injection procedure, was significantly shorter with CARTBox2 than with NOGA. Substituting the mapping time in our study with the typical value of 45 min still leads to a significantly shorter total procedure time for CARTBox2 (69.2 ± 11.9 min) compared to NOGA (103.2 ± 13.6 min; p = 0.004).

In contrast to NOGA, CARTBox2 requires the acquisition (1 h) and segmentation (15 min) of an LGE-MRI scan. Since both are performed prior to the injection procedure, Cath Lab procedure time is not increased by CARTBox2. The 3D-RA roll scan required for CARTBox2 is acquired prior to the injection procedure and takes less than 1 min but does add to the total amount of radiation of the CARTBox2 procedure. Subsequently, registration of the LGE-MRI with 3D-RA was performed during the time in which the arterial access sheaths were placed, and consequently did not increase the procedure time. In this study, we found no significant difference in the time required for the intramyocardial injections of both systems. Of note, the targeting facilitated by CARTBox2 is of a different nature compared to NOGA. With CARTBox2 the physician is aiming for a single target at a time, without visualization of the IBZ or neighboring targets (Fig. 3b, c), whereas with NOGA the physician can navigate over the IBZ, whilst other injections are visualized (Fig. 3a). The abovementioned fundamental difference between both systems are likely to have caused an increase of the procedure time of the CARTBox2 procedures.

### XRF time and dose

Injections performed with CARTBox2 required significantly more XRF time in comparison with the NOGA system. The DAP per minute of XRF per injection is lower in the CARTBox2 group, showing that the XRF angulation and rotation used with CARTBox2 (mainly right/left anterior oblique) required less XRF energy compared to the angulation used with NOGA (mainly anterior-posterior). With the NOGA system being a non-fluoroscopic navigation system, XRF guidance is only required for passing the aortic valve and during the initial mapping points. We were unable to find any reports in the literature on XRF usage during NOGA procedures. The average reported doses for a PCI are in the range of 70–150 Gy cm^2^ [17, 18]. Data of the present study showed that the XRF dose with CARTBox2 procedures is similar or lower than an average PCI.

### Ventricular arrhythmia monitoring

In the present study 2/5 and 1/5 animals required cardioversion during the NOGA and CARTBox2 procedures, respectively. The number of non-sustained ventricular arrhythmias per injection was similar in both study groups, suggesting that the longer procedure time (mapping) of NOGA may be responsible for the higher total number of ventricular arrhythmias. However, due to the large variation in PVCs in the NOGA group and with non-sustained VTs in both groups, no significant differences were observed between both groups.

### Limitations

In this study a single plane XRF device was used. The use of a biplane XRF device obviates the need for multiple C-arm rotations to verify optimal catheter positioning, saving much procedure time. It is therefore likely that the total procedure time with CARTBox2 would have been considerably shorter if a biplane XRF setup had been used.

The CARTBox2 approach of visualizing the target as a single point instead of a targeting line, forced the operator to maneuver the catheter to that exact position. This approach inevitably increased injection duration during the CARTBox2 procedure when a target was difficult to reach. Instead of changing focus to a reachable target, time was spent on reaching difficult targets. The visualization of a single target at a time by CARTBox2 was chosen to optimize the 3D orientation of the physician and ease of navigation by XRF using a single plane fluoroscopy system. Showing multiple targets or the complete IBZ in combination with increased user experience will further reduce the time required for the CARTBox2 procedure.

Since the NOGA map is constructed from points that are acquired on the LV endocardium using a catheter, the target region is always reachable with the catheter. In contrast, targets selected with CARTBox2 are selected manually from LGE-MRI and can be difficult to reach with the catheter due to for example the presence of papillary muscles or the basal location of the injection target. More experience with the pre-procedural selection of injection targets can prevent selection of targets that are difficult to reach.

### Clinical implications

In the present study we have compared CARTBox2 to a clinically available electromechanical mapping system for intramyocardial injections into the IBZ. CARTBox2 is however not limited to this type of procedures and it can be used for all procedures for which pre-operative treatment planning based on 3D imaging data is beneficial. Other possible applications are lead placement for cardiac resynchronization therapy devices, acquiring endomyocardial biopsies or performing ablations in regions with a scar substrate. The fusion of processed LGE-MRI data, or wall thickness analysis from multi-detector CT scans with the electro-anatomical navigation systems to guide ventricular tachycardia substrate mapping and ablation have been described [19, 20].

With CARTBox2, not only targets can be identified, but also areas that should be avoided can be annotated. In the case of intramyocardial injections or endocardial biopsies such areas may include sections in which the myocardium is too thin, in case of lead placement, such areas may include stretches of nonconductive fibrous myocardium or locations in proximity to the phrenic nerve.

For the purpose of intramyocardial injections, the use of CARTBox2 as an alternative to the NOGA system for intramyocardial injections may significantly shorten the procedure time and increase Cath Lab capacity. Furthermore, CARTBox2 obviates the need for expensive dedicated intramyocardial injection systems in terms of acquisition, training and operating (e.g. mapping and injection catheters) costs. The radiation dose with CARTBox2 injection procedures is higher than during the NOGA procedures, but does not exceed that of a typical PCI, and is likely to decrease with experience.

## Conclusion

CARTBox2 is a safe and accurate alternative ‘software-only’ technology for treatment planning and fluoroscopy based image guided cardiac catheter therapies. The data of this study on the specific topic of intramyocardial injections shows that the procedures performed with CARTBox2 are equally accurate and quicker. In contrast, CARTBox2 requires acquisition of an LGE-MRI scan and requires more fluoroscopy than NOGA. The software only CARTBox2 method enables its use in all cardiologic centers that have modern fluoroscopic imaging equipment.

## Electronic supplementary material

Below is the link to the electronic supplementary material.

Supplementary material 1 (DOCX 15 KB)

## Abbreviations

BV: Bipolar voltage
Gy: Gray (SI unit; J/kg)
IBZ: Infarct border zone
I/R: Ischemia/reperfusion
LGE: Late gadolinium enhanced
LV: Left ventricle
MRI: Magnetic resonance imaging
PCI: Percutaneous coronary intervention
PVC: Premature ventricular contraction
VT: Ventricular tachycardia
XRF: X-ray fluoroscopy
