# Supplementary material for: Validation of a novel stand-alone software tool for image guided cardiac catheter therapy
Source: Int J Cardiovasc Imaging. 2019 Jan 28;35(2):225–35. doi: 10.1007/s10554-019-01541-9 (PMC6428788; doi:10.1007/s10554-019-01541-9)
Supplement: Supplementary file 1 — Supplementary material 1 (DOCX 15 KB) [file 10554_2019_1541_MOESM1_ESM.docx]

Supplemental Material

## MRI parameters (pre-injection)

### Cine

Repetition time [TR]/echo time [ET] = 3.39 ms/1.69 ms. Flip angle = 60 °, Pixel size = 1.25 x 1.25 mm, [FOV] = 320 x 320 mm, 256 x 256 matrix, 8 mm slice thickness.

### Late Gadolinium Enhancement

[TR]/[ET] = 3.64 ms/1.19 ms. Flip angle = 25 °, Pixel size = 1.25 x 1.25 mm, [FOV] = 296 x 296 mm, 256 x 256 matrix, 4 mm slice thickness.

## MRI parameters (ex-vivo)

### LGE

[TR]/[ET] = 5.53 ms/1.69 ms. Flip angle = 25 °, Pixel size = 1.0 x 1.0 mm, [FOV] = 169 x 169 mm, 176 x 176 matrix, 3 mm slice thickness.

### T2*

[TR]/[ET] = 88.7 ms/15 equally distributed ETs with range 1.9-24.6 ms. Flip angle = 15 °, Pixel size = 0.5 x 0.5 mm, [FOV] = 169 x 169 mm, 320 x 320 matrix, 3 mm slice thickness.

## XRF parameters

Cardiac low dose, 3,75fps. Exposure prefilter 0.10 mm Cu + 1.00 mm Al.

## 3D-RA parameters

180° rotation in 10.2s, 30fps. Max rotation speed 30°/s. Tube voltage 125kV. 128x128 reconstruction matrix
